# A synergistic unsupervised-supervised learning approach for data-driven and clinically applicable patient phenotyping

**DOI:** 10.1016/j.ebiom.2024.105358

**Published:** 2024-09-20

**Authors:** Mert Karabacak, Konstantinos Margetis

**Affiliations:** Department of Neurosurgery, Mount Sinai Health System, New York, NY, USA

We read with great interest the article by Pedro et al. identifying four unique phenotypes of degenerative cervical myelopathy (DCM) patients.^1^ The authors' innovative use of unsupervised machine learning (ML) techniques represents a significant advancement in our understanding of DCM phenotypes.

While the study provides valuable insights, we believe there is an opportunity to further enhance its clinical applicability. The current approach, while robust, may present challenges in determining the specific cluster to which an individual patient belongs. For instance, a patient with a high neck pain score but low motor impairment might not clearly fit into any single phenotype based on the provided mean scores. This potential limitation could hinder the practical application of this well-thought approach in clinical settings and further research.

To address this, we propose a multi-step approach to develop a clinically applicable classification method:

1. Train multiple explainable, rule-based classification models to predict cluster membership. We suggest using interpretable decision tree-based classifier (e.g., CART,^2^ C4.5^3^^,^^4^) or rule-based classifier (e.g., RIPPER^5^) algorithms for this purpose. The models created using these algorithms should offer clinicians easily applicable rules for determining the cluster membership of individual patients in their practice or research.
2. Measure the performance of these prediction models in accurately assigning patients to their original clusters.
3. Analyze and publish the decision tree or rules generated by the best-performing model to gain insights into the key factors driving cluster membership. A balance between model complexity and performance is required based on the authors' judgement.

This approach offers several advantages. First, it allows for classification of new patients in the clusters the authors found in the study found. Secondly, it provides a more accurate representation of how the clustering algorithm determined the clusters, potentially revealing insights beyond what can be inferred from mean values alone. The decision trees or rules would explicitly show the combination of factors and their thresholds that lead to each cluster assignment, offering a more nuanced understanding of the phenotypes. This method addresses the potentially misleading nature of relying solely on mean values to characterize clusters. By directly modeling the decision boundaries between clusters, we can capture more complex relationships in the data that might not be apparent from summary statistics. Lastly, the resulting classification rules would be easily interpretable and applicable by clinicians, bridging the gap between sophisticated machine learning techniques and practical clinical use.

This approach combines the strengths of unsupervised and supervised ML techniques while addressing the need for practical, reproducible patient classification. It could significantly enhance the clinical utility of the authors' work and facilitate its adoption in both research and practice.

## Contributors

Conceptualisation, MK and KM; Writing–Original Draft, MK; Writing–Review & Editing, KM.

## Declaration of interests

None.
